# Clinical Features and Outcomes of Treatment for Effusive Feline Infectious Peritonitis with GS-441524 in Seventeen Retrovirus-Positive Cats

**DOI:** 10.3390/pathogens15030337

**Published:** 2026-03-21

**Authors:** Marilize Van der Walt, Sarah E. Jones, Julie K. Levy, Emma Hart, Rosa Negash, Wendy M. Novicoff, Nicole Jacque, Samantha J. M. Evans

**Affiliations:** 1 Internal Medicine Department, MedVet Medical & Cancer Center for Pets, Columbus, OH 43085, USA; sarah.e.jones@medvet.com; 2 Shelter Medicine Program, College of Veterinary Medicine, University of Florida, Gainesville, FL 32608, USA; levyjk@ufl.edu; 3 Faithful Friends Veterinary Clinic, Dublin, OH 43017, USA; dr.hart@faithfulfriendsvc.com; 4 Heritage College of Osteopathic Medicine, Ohio University, Athens, OH 45701, USA; rn488016@ohio.edu; 5 Departments of Orthopaedic Surgery and Public Health Sciences, School of Medicine, University of Virginia, Charlottesville, VA 22901, USA; wmn2v@uvahealth.org; 6 Independent Researcher, San Jose, CA 95123, USA; jacque@mac.com; 7 Department of Microbiology, Immunology and Pathology, Colorado State University, Fort Collins, CO 80523, USA; samantha.evans@colostate.edu

**Keywords:** feline infectious peritonitis, GS-441524, feline leukemia virus, feline immunodeficiency virus, treatment

## Abstract

Background: There is limited information about treatment success and outcomes in retrovirus-positive cats diagnosed with feline infectious peritonitis (FIP). Methods: A survey was distributed to caretakers of cats with feline leukemia virus (FeLV) and/or feline immunodeficiency virus (FIV) that were treated with GS-441524 for presumptive effusive FIP based on survey responses. Results: Cats with FIV developed FIP at an older age and longer after retrovirus infection than cats with FeLV. The average starting dosage (7 mg/kg/d) was increased in 65% of cats, and treatment was extended in 35%. Three cats relapsed (18%). There was a 94% (16/17) twelve-week survival rate and 82% (14/17) one-year survival rate. Seven cats were alive at follow-up, a median of 1306 days (range 983–2069) after FIP diagnosis, but many cats succumbed to neoplasia. Conclusions: Treatment success for retrovirus-positive cats with presumptive FIP was similar to previously reported outcomes for FIP alone. This could support current evidence of successful antiviral therapy for similar populations, if noncurrent, unstandardized protocols and unlicensed product use are considered. Additional studies are needed to determine ideal protocols for rapid resolution of FIP, good long-term survival, and limited relapse in retrovirus-positive cats, and the impact of the FeLV proviral load.

## 1. Introduction

Important viral causes of morbidity and mortality in cats include feline infectious peritonitis virus (FIPV), and the retroviruses feline immunodeficiency virus (FIV) and feline leukemia virus (FeLV). FIPV is a virulent biotype of feline coronavirus (FCoV) that is thought to arise upon acquisition of mutations during FCoV infection [1,2]. FCoV infection itself is usually self-limiting, though persistent and recurrent infections have been described, with viral shedding lasting years [3]. Feline infectious peritonitis (FIP) is characterized by the formation of immune complexes, which, depending on the degree of antibody-mediated vs. cell-mediated involvement in the immune response, lead to the development of disease predominated by cavitary effusions (“wet” or effusive FIP) or granulomatous inflammatory lesions (“dry” or non-effusive FIP) [4]. Without treatment, FIP is uniformly fatal [5]. Infections by retroviruses FIV and FeLV are characterized by immune dysregulation and immunosuppression via T lymphocyte deficiencies and dysfunction [6], and cause mortality from secondary infections, bone marrow dyscrasias, and neoplasia [7,8,9]. As a result, cats with FeLV have significantly shorter survival than FeLV-negative cats [8].

There is an interplay between the development of FIP and infection with a retrovirus. FIP is diagnosed twice as commonly at necropsy in cats with FeLV [7]. Before the advent of FeLV test-and-segregate control programs in the late twentieth century, up to half of all cats with FIP were also infected with FeLV [5]. FIV infection also affects the response to FCoV. A previous study [10] found that chronic FIV-positive cats experimentally infected with FCoV shed the virus in their feces for longer and to a greater magnitude than FIV-naive cats. These FIV-positive cats also had delayed development of FCoV antibodies and lower serum titers. These factors were suspected to contribute to a higher risk of development of mutations causing FIPV.

Until recently, there was no effective treatment for FIP [11]. Antiviral drugs containing GS-441524, a nucleoside analogue, and related compounds were first reported to be effective in naturally infected cats in 2019 and have since reached global use with survival rates of 80–100% [12,13,14,15,16]. However, the drugs were not yet approved in many countries, so cat caregivers sought aid through online networks that provided information on how to source unlicensed GS-441524 as well as protocols for treatment and monitoring of FIP. This generally consisted of 12 weeks of antiviral administration, followed by a 12-week observation period. The cost of the full course of treatment using crowd-sourced GS-441524 was expensive, with owners reporting a mean of 4920 USD in a previous study by our group [17]. GS-441524 and other antiviral medications became available in 2024 through licensed compounding pharmacies in the United States, after which regulated compounded formulations became available at a much lower cost (less than 1000 USD for most cats in 2025).

A previous report postulated that the immunosuppression caused by FeLV might interfere with the effectiveness of antiviral therapy for FIP in co-infected cats [18]. The report further suggested that the average shorter lifespan of cats infected with FeLV might offset the value of treating them for FIP when the complications and cost associated with obtaining and administering antiviral drugs are taken into account. However, a recent study found that a cohort of 104 FeLV-positive cats had a similar response to therapy for FIP compared to FeLV-negative cats, but the overall survival time was still cut short in FeLV co-infected cats [19]. Another study on the interactions of multiple viral co-infections with FIP reported a low prevalence of FeLV (2%) and FIV (4%) and a treatment response rate equivalent to cats without FeLV or FIV [20]. Beyond that, little information is available in the peer-reviewed literature on the FIP treatment success in retrovirus-positive cats. The aim of this study was to describe caregiver-reported clinical features and outcomes in a convenience sample of FIV- and/or FeLV-positive cats presumptively diagnosed with FIP, and treated with GS-441524.2.

## 2. Materials and Methods

A survey (Supplementary Materials) using the Qualtrics software platform (Version September 2021, Qualtrics, Provo, UT, USA) was distributed to social media plat-forms used to aid in the treatment of cats with suspected FIP, as well as to animal shelters with known cases of FIP. The survey was targeted toward caretakers of retrovirus-positive cats who were treated for suspected effusive FIP. Effusive FIP was suspected to be present when supporting clinical signs and cavitary effusion were reported, or a diagnosis was reported to be made by a veterinarian. Provision of veterinary records was not required, so FIP and retrovirus diagnosis relied on self-reported diagnostic data. A full work-up for FIP included a CBC, serum biochemistry, imaging (abdominal ultrasound and/or radiographs), analysis of effusion (complete fluid analysis, cytology, and/or Rivalta), and FIP-specific testing (FCoV RT-PCR, FIP mRNA PCR, FIPV RealPCR, and/or ELISA for 7b protein). Due to diagnostic uncertainty, a confidence-based framework was retrospectively applied. Cats were considered “low confidence” for FIP diagnosis if the diagnosis was suspected based solely on CBC, chemistry, and/or imaging (i.e., there was no testing performed on the effusion, nor FIP-specific testing). As results of FeLV-specific diagnostics were not available, the FeLV stage of infection could not be obtained, becoming a core limitation to the interpretation of our results.

Inclusion criteria were cats reported to have positive FeLV and/or FIV test results and treatment plans for at least 12 weeks of GS-441524. One cat did not complete all 12 weeks due to progression of disease leading to death but was included due to the original planned length of treatment. Survey questions were adapted from a survey previously distributed by our group [17], with additional questions incorporated regarding retroviral disease. The draft survey was tested by two veterinarians and three cat caregivers. The final survey took approximately 20–25 min to complete. Informed consent was obtained, and this study was approved by the Institutional Review Board (protocol #2021E0162) at the Ohio State University. Questions were either multiple choice or short answer format, and some responses triggered the presentation of additional questions. Final surveys were excluded where a majority of the survey was incomplete, or for cats that were treated for non-effusive FIP. Final survey submissions occurred between 4 March 2022 and 8 June 2023. Follow-up correspondence occurred between 19 May 2025 and 29 October 2025 via email inquiring about unanswered questions and the current status of their cat.

During data analysis, when exact dates for diagnosis of infection and survival were not available, dates were estimated and truncated to the first of the month or the following month when appropriate. Descriptive statistics and frequencies for categorical values were determined using Google Sheets (version 2025, Google LLC, Mountain View, CA, USA). Statistical analysis was performed using Minitab (Version 22.1, Minitab, LLC, State College, PA, USA). The Mann–Whitney test with an alpha level of 0.05 was used to compare age-related parameters of the FIV and FeLV groups, and a Kaplan–Meier curve was used to compare survival between groups. For the latter, data were censored if the cat was still alive at follow-up or was lost to follow-up (i.e., cats without known or estimated dates of death).

## 3. Results

### 3.1. Demographics

A total of 26 survey respondents consented to being included in this study. Nine were excluded due to a presumptive diagnosis of non-effusive FIP. Seventeen cats were included in the final analysis. Follow-up responses were available for 14 cats over an average of 776 days (range 711–800 days) after initial survey submission. Cats AI, BI, and NL were lost to follow-up after the initial survey submission, but survival could be estimated for AI. For brevity, respondents (*n* = 26) ⟶ excluded (9 non-effusive) ⟶ included (17) ⟶ follow-up available (14). For ease of reference, cat IDs referenced in the text are formatted to include the retrovirus status (i.e., “I” for FIV, “L” for FeLV, and “IL” for co-infection).

Most respondents were located in the United States of America (*n* = 14), with one cat each from Italy, Brazil, and Portugal. There were 14 domestic mixed-breed cats, two American Shorthairs, and one Common European. There were four spayed females, and 13 males, of which 11 were neutered and two intact. Caregivers reported that nine cats were FeLV-positive, six were FIV-positive, and two cats were both FeLV- and FIV-positive.

The characteristics of FIP, median ages, times between diagnosis of a retrovirus and FIP, and survival data are listed in Table 1. The median age of diagnosis and range for each infection by group are described in Table 2. The age of diagnosis of FIP in cat DI (267.3 months) was confirmed via follow-up communication, though the cat’s age was likely estimated at the time of rescue. Due to the methodology used and available data, one cat was listed as diagnosed with FeLV at birth.

**Figure 1:**
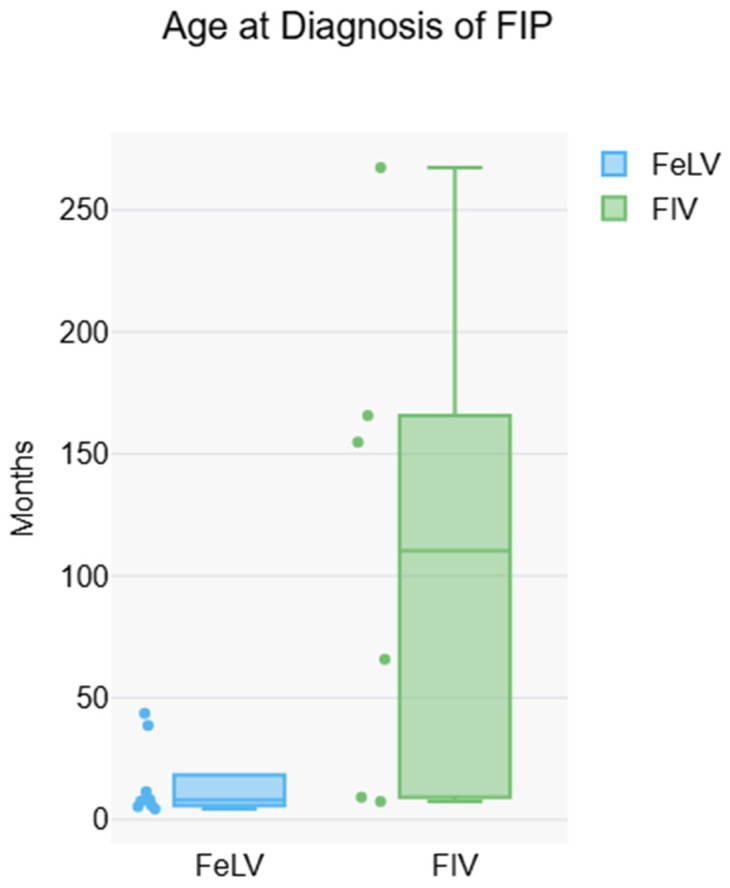
Comparison of age at diagnosis of FIP in FeLV-only and FIV-only cats, including cats diagnosed with a retrovirus and FIP at the same time. FeLV-only cats were significantly younger (*p* = 0.036, *n* = 15).

Exploratory analysis on ages, disease characteristics, and chronology was performed. Most cats with FeLV-only (*n* = 9) were younger than cats with FIV-only (*n* = 6), though this difference was not statistically significant, likely reflecting limited power (*p* = 0.052, *n* = 15). Cats with FeLV-only were younger at diagnosis of FIP than cats with FIV-only, both including (*p* = 0.036, *n* = 15) and excluding (*p* = 0.010, *n* = 12) cats whose retroviruses were diagnosed at the same time as FIP (Figure 1). The median time between diagnosis with a retroviral infection and diagnosis of FIP was shorter for FeLV-only cats than FIV-only cats only if cats whose retrovirus was diagnosed at the same time as FIP was excluded (*p* = 0.010, *n* = 12).

At the time of FIP diagnosis and during treatment, 11 were privately owned, and 6 were under the care of a rescue group or shelter. Seven cats were adopted as strays, while all but two of the rest were adopted from a rescue group (*n* = 4) or shelter (4). For the remaining two cats, the adoption history of one could not be determined from the provided answer—the cat was described as “rescued” but not from where—and information for the other cat was unavailable due to a blank survey question and loss to follow-up. At the time of FIP diagnosis, respondents had cared for the cat for less than one month (1), between one and six months (6), between six months and one year (3), between one year and two years (1), between two years and five years (3), five years or more (2), or did not provide an answer (1). Both cats that were owned for five years or more had FIV.

### 3.2. Clinical Signs

Common clinical signs that occurred around the time of FIP diagnosis are listed in Table 3. Clinical signs that occurred in fewer than 20% of all cats include anisocoria, cough, diarrhea, upper respiratory signs, lymphadenomegaly, cardiac disease, icterus, increased water consumption, ocular discoloration, constipation, urinary incontinence, paralysis, and pica.

Three cats were diagnosed with ocular FIP, and two with concurrent neurologic FIP (JL, ML, NL). Reported ocular changes in cats with ocular FIP included color changes or spots in the eye (NL), elevated third eyelids (ML), and uveitis (ML). Neurologic FIP was initially diagnosed in two cats (JL, NL). Presenting neurologic signs included one cat with anisocoria (NL), two cats with difficulty walking or jumping (JL, NL), and one cat with partial or total paralysis (JL). During treatment with GS-441524, respondents of three additional cats (FI, GL, OL) who were not originally diagnosed with neurologic FIP commented on the development of neurologic signs. These included ataxia (FI) and seizure activity (FI, OL), occurring as soon as the first week of treatment (FI). Cat JL, who was diagnosed with neurologic FIP at the time of FIP diagnosis, developed worsening neurologic signs (gait changes) two months after completing 36 weeks of GS-441524 therapy. An MRI showed marked hydrocephalus and syringohydromyelia. There was no evidence of active FIP infection, but the changes were suspected to be associated with the previous FIP infection. A congenital malformation could not be excluded.

### 3.3. Diagnosis

A veterinarian diagnosed FIP in 14 cases, either their own veterinarian (*n* = 11) or a shelter/rescue’s (3). Three cats were diagnosed with FIP online using the diagnostic results from a veterinarian (GL, JL, OL). All three caretakers discussed this suspected diagnosis with their veterinarian, who in turn referred two of them to an online group for continued treatment (JL, OL) or were involved with ongoing monitoring with the other (GL).

Diagnostics performed for each cat are listed in Table 4. Two cats had a full work-up. Including these two, FIP-specific testing was performed in five cats. None of the three cats diagnosed online had FIP-specific testing performed. Six cats (BI, DI, GL, KL, LL, and OL) were considered “low confidence” for FIP diagnosis, with diagnosis suspected solely on CBC, chemistry, and/or imaging (i.e., there was no testing performed on the effusion, nor FIP-specific testing). Two of these cats (BI, and GL, who was diagnosed online) provided supplemental lab work, which showed elevated serum globulins and an albumin-to-globulin ratios of 0.3 at the time of FIP diagnosis. All cats received at least one diagnostic requiring veterinary involvement. In all but one cat (GL), the effusion was aspirated and examined grossly.

The most common clinicopathologic abnormalities commented on included anemia (*n* = 6) and neutropenia (2). When effusion fluid description was reported (13), it was commonly yellow-tinged (9), viscous, sticky or thick (5), cloudy (3), clear (2), green-tinged (1), opaque white with a “broken glass” appearance (1), or red/pink-tinged (1). Five respondents reported the fluid being a modified transudate and/or having a high protein content. Imaging findings were sometimes provided and included irregularly shaped kidneys and thickened intestines on abdominal ultrasound (BI), and a mediastinal mass effect on radiographs that was diagnosed as a diaphragmatic hernia (JL). The latter cat also had an MRI performed, as previously described.

Cats tested positive for retroviruses before the development of FIP in 10/11 FeLV-positive cats and 7/8 FIV-positive cats, including both co-infected cats. One cat with FeLV (OL) and one cat with FIV (CI) were diagnosed when the cat started showing clinical signs of FIP. When FeLV was diagnosed prior to FIP, it was by respondents’ personal vet who tested for it as part of a routine wellness care (*n* = 2), after exposure to a FeLV-positive cat (1), or upon diagnosis of illness other than FIP (1). The other five FeLV-positive cats were tested at a shelter/rescue (2), or respondents were told upon adoption that the cat was positive (3). Of seven cats diagnosed with FIV prior to FIP diagnosis, three were diagnosed when they were due to be spayed/neutered, three had already been diagnosed before adoption, and one was diagnosed at a health/wellness visit. Four FeLV-positive cats were known to be vaccinated for FeLV, six were not vaccinated, and one had an unknown vaccination history. Six of the eight FIV-positive cats had not been previously vaccinated and the other two had unknown vaccine histories.

### 3.4. Treatment

Most (10/17) respondents’ veterinarians informed them that there was a treatment available and referred respondents to another organization for more information. Others explained the treatment in detail (*n* = 3), explained that treatment was not available (1), or that treatment was available, but they were not knowledgeable about it (1). Two respondents were told that there was no curative treatment. Several (5/17) respondents heard about GS-441524 therapy from multiple sources. When respondents learned about GS-441524 from someone in animal healthcare, it was either their general practice veterinarian (9), their shelter/rescue group (5), a veterinary specialist (3), or through their own occupation in veterinary medicine (1). External sources included the internet or social media (4), or a friend or family member (2). After diagnosis, thirteen respondents had veterinary involvement for monitoring, with one respondent’s veterinary team administering the medication. This cat (CI) lived in Italy. Four respondents proceeded without assistance from a veterinarian after diagnosis.

Sixteen cats completed at least 12 weeks of treatment. One cat (HL) did not survive this period and only completed eight weeks of treatment before the development of acute lymphocytic leukemia, suspected to be secondary to the progression of FeLV. The median length of GS-441524 therapy was 12 weeks (range 8–36). Treatment was extended beyond the initial 12 weeks in six cats for a median of 5.5 additional contiguous weeks (range 3–24 weeks beyond the initial 12-week period). Reasons for extension included blood work findings (*n* = 4), ongoing clinical signs (3), concurrent retroviral infection (2), or as a general precaution (1). Only one of the four cats that had treatment extended as a result of blood work findings was also showing clinical signs (clinical signs not specified). Three cats relapsed (Table 5). Cat JL was treated for an additional 24 weeks beyond the initial treatment period, the first additional 12 because of ongoing clinical signs, and the second 12 because of lab work abnormalities. This cat had only received injectable GS-441524 before the first relapse. The other two cats that relapsed (ML, OL) were also showing clinical signs, which prompted dose increases.

The average reported starting and ending dosages for the 14 cats with data available are listed in Table 6. Three respondents were unable to remember the dosages. All cats were started on injectable GS-441524. The protocol remained the same for five cats for both dosage and drug form in the initial 12-week period. One of these cats (OL) had a dose increase after restarting therapy for a relapse and neurologic signs several weeks later. At least 11 cats experienced dosage increases at some point during therapy. These cats included the cat above who relapsed, one of the three cats with unknown dosages, and two cats whose end dosages in Table 5 were lower than the reported starting dosages. Reasons for increase in these 11 cats included the development of neurologic signs (3), lab work findings (*n* = 2), retroviral status (1), clinical signs (1), and recommendations by social media administrators (1). Three did not specify, but two of these had been switched to oral medications. In total, seven cats were switched to oral therapy, but one cat (GL) returned to injectable after a poor response following the switch. Reasons for the switch included injection site reactions. With respondents residing in the United States, Italy, Brazil, or Portugal prior to the availability of legally prescribable compounded GS-441524, these cats are presumed to have received unlicensed GS-441524 only.

In summary, the average starting dose of 6.8 mg/kg/d was escalated in at least 11/17 (65%) cats. This included 4/5 cats with neurologic signs, 2/3 cats with ophthalmologic signs (with one cat common to both groups), and 3/3 cats that relapsed. Seven cats were switched to oral therapy. The median length of GS-441524 therapy was 12 weeks, and treatment was extended for six cats for a median of 5.5 additional weeks.

One cat received the additional antiviral Raltegravir (IL) during GS-441524 therapy. Twelve cats received antibiotics, including amoxicillin clavulanate (*n* = 5), cefovecin (2), doxycycline (2), unspecified (2), marbofloxacin (1), azithromycin (1), and topical ophthalmic (1) or otic (1) solutions. Other treatments included gabapentin (9), antiemetics (8), subcutaneous fluids (5), subcutaneous cobalamin (4), oral steroids (4), intravenous fluids (2), mirtazapine (2), NSAID (1), blood transfusion (1), buprenorphine (1), intravenous albumin (1), iron supplement (1), and supplemental oxygen (1). Supplements included T-cyte/Proboost/Thymic Protein A supplementation (3), lysine (1), and Polyprenyl Immunostimulant or VetImmune (1). One cat received vincristine for lymphoma after treatment of FIP.

### 3.5. Concurrent Illness

Diagnoses that occurred prior to FIP diagnosis included diabetes (PIL), pododermatitis (QIL), and a peritoneo-pericardio-diaphragmatic hernia that was surgically corrected (JL). The latter cat also had congenital ocular abnormalities, a previous enucleation, and glaucoma, which was being medically managed with topical ophthalmic drops. Cat EI was historically diagnosed with congestive heart failure and had pulmonary edema. This cat had a history of dynamic left ventricular outflow tract obstruction and moderate hypertrophic cardiomyopathy. This cat also had a bronchointerstitial pattern on radiographs and was diagnosed with lower airway disease (asthma). These conditions were being medically managed with fluticasone, furosemide, and clopidogrel during treatment. This cat also had periodontal disease that was managed chronically with extractions and prophylactic cleanings.

Concurrent illnesses diagnosed around the time of FIP diagnosis included an ear infection (cat AI) for which it was receiving a topical ointment containing thiabendazole, dexamethasone, and neomycin sulfate. Cat DI had mycoplasma (unspecified species) and received both azithromycin and doxycycline during FIP treatment. Cat PIL had an upper respiratory tract infection.

Illnesses that occurred after treatment of FIP and after the observation period but before the time of the survey included inflammatory bowel disease in cat DI, and calicivirus in cat AI. Additional illnesses provided post-survey at the time of follow-up are discussed below.

### 3.6. Outcomes

**Figure 2:**
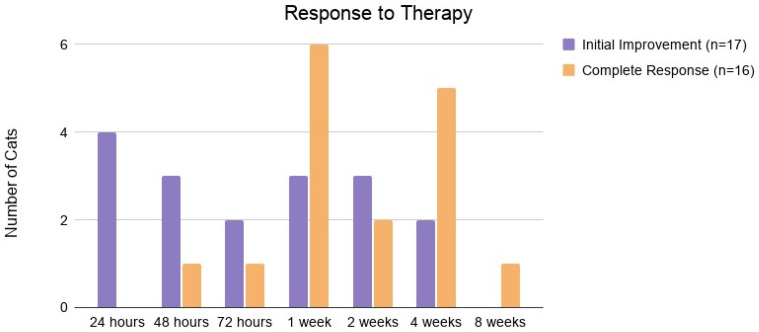
Time to initial improvement in clinical signs or fully normal behavior after starting treatment.

Time to initial improvement and complete resolution of clinical signs is shown in Figure 2. One cat with both neurologic and ophthalmologic FIP (JL) never fully returned to normal, with progressive deterioration of vision and persistent gait abnormalities. The median weight cats gained was 1.0 kg (range −0.4 kg to 4.5 kg).

At the time of taking the survey, 12 cats had finished the observation period, and 1 cat was within the 12-week observation period (GL). Of the 12, 9 had finished the observation period over one year earlier, including all 3 cats that had relapsed. Four cats were reported to have died or been euthanized at the time of the survey (Table 1). Three of these were deceased due to lymphoid neoplasia, including cat EI who had developed a jejunal mass and was euthanized within two months after poor response to vincristine. The fourth cat (AI) survived at least nine months after FIP diagnosis but was not alive at the time of the survey (two years later). This cat’s decline in condition was not reported to be due to neoplasia, but more information was not provided.

In addition to the four cats that were deceased at the time of the survey, four more cats were deceased at the time of follow-up (Table 1). Cat PIL lived “a couple years” after going into remission for FIP before developing an unspecified gastric tumor. Cat JL developed a perforated small intestine with a septic abdomen and was euthanized 845 days after FIP diagnosis. The necropsy findings were suggestive of an infiltrative neoplastic process such as chronic eosinophilic leukemia or other round cell neoplasia with additional factors including pancreatitis, bile duct obstruction, and sepsis contributing to this cat’s decline and ultimate euthanasia. Cat KL lived “over a year” after FIP diagnosis and succumbed to complications suspected to be related to the FeLV rather than FIP, though specific details were not provided. Cat QIL was reported to be in excellent health three years following completion of treatment for FIP. This cat developed a suspected aortic thromboembolism with acute fecal and urinary incontinence, and treatment was started for possible neurologic FIP. Three weeks later, ocular lymphoma was diagnosed and the cat developed progressive tetraparesis and seizures. The cat was ultimately euthanized within the following six months due to poor quality of life.

**Figure 3:**
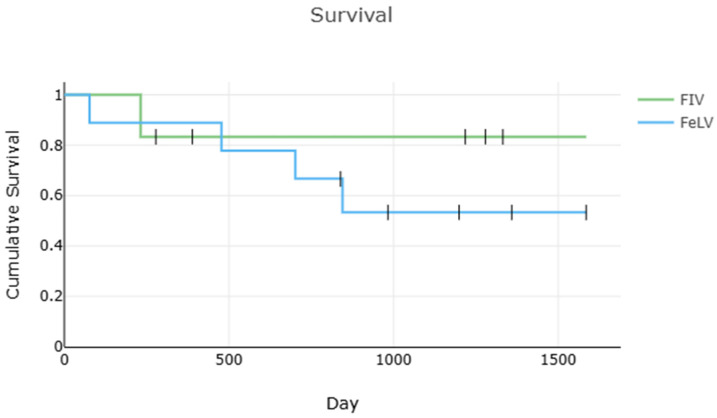
Survival for cats with FeLV-only and FIV-only using known and estimated dates. There was no significant difference between groups (*p* = 0.47, *n* = 15).

Survival data is displayed in Figure 3. Dates include estimates/truncations for some cats (Table 1). The overall twelve-week survival rate was 94% (16/17), and the one-year survival rate was at least 82% (14/17). When low confidence cats were excluded, the survival was 91% (10/11), and the one-year survival rate was at least 73% (8/11). A total of 14 cats were available for follow-up; of these, 7 were still alive a median of 1279 days (range 983–1585) after diagnosis of FIP. The median survival after FIP diagnosis for all cats was 845 days (range 77–1585), which remained unchanged when low-confidence cats were excluded. The median survival of cats with FIV-only was 804 days (range 232–1332), and 845 days (range 77–1585) for FeLV-only. There was no significant difference in survival between cats with FeLV-only and FIV-only (*p* = 0.470, *n* = 15), though low power limits this interpretation.

All 17 respondents were “very satisfied” with their experience of undergoing GS-441524 therapy for their cat. When asked how likely respondents would be to undertake GS-441524 therapy in the future, 15 responded “very likely,” one “neutral,” and one “unlikely.” The respondents who listed “neutral” and “unlikely” clarified that this was because of the financial and emotional stress surrounding the process. Along with these two respondents, two other respondents also commented on the financial challenge. One respondent specified that they would treat a non-FeLV cat again. The additional comments provided were enthusiastic and passionate, and many expressed gratitude for the extra time they now had with their cat.

## 4. Discussion

This study presents the findings of a survey investigating the clinical features and outcomes of 17 retrovirus-positive cats that were treated with GS-441524 for presumptive FIP based on survey responses. All cats seemingly responded to the antiviral therapy, and at least 14 of the 17 cats survived at least one year after beginning treatment for suspected FIP. Six cats that failed to fully respond to the standard 12-week treatment period had medication extensions up to an additional 24 weeks. Three cats were suspected of having suffered relapse of FIP within four weeks of completing the initial treatment plan but improved after dose escalations. Though the limitations described in this report should be considered, these findings are consistent with the possibility that antiviral treatment can be successful in these populations. The initial survey was conducted in 2022–2023, prior to the availability of regulated GS-441524 in the US and widespread veterinary awareness of its use. As a result, cat caregivers relied on the importation of GS-441524 of uncertain quality and concentration, treatment guidance by volunteers in social media groups, and dosing levels that were approximately half of currently recommended amounts [21]. This is likely why there were great differences in dosage regimens in our report. Current recommendations for treatment are to start with a validated source of GS-441524 dosed at 15 mg/kg/d for 42 [22] to 84 days [23], followed by an 84-day observation period during which signs and laboratory values are monitored for normalization. Dose escalation is recommended to 20 mg/kg/d for neurological, ocular, and relapse conditions. It is possible that long-term outcomes could have been improved had this updated protocol been available at the time of treatment of the cats in the current study.

The clinical characteristics of suspected FIP included in our study were frequently similar to previous reports in studies with confirmed cases of FIP. These comparisons are tentative, due to our less stringent inclusion criteria for diagnosis of FIP. The most common clinical signs were often nonspecific indicators of disease and included hyporexia or anorexia, lethargy or listlessness, and weight loss. Another common clinical sign was a distended abdomen. This corresponded to the development of abdominal effusion in most cases, which occurred in 82% of effusions. This clinical presentation is similar to other studies with confirmed cases of FIP [20,24,25], which found lethargy, depression, inappetence, fever, and weight loss to be the most common clinical signs, and found ascites comprised 77–84% of effusions. These clinical signs are also similar to other reports of at-home treatment for FIP [17].

Of the seventeen cats, two (12%) had neurological signs at the time of FIP diagnosis and three others developed neurological signs during or after treatment (three of fourteen with follow-up; 21%). This proportion of cats with neurologic clinical signs is similar to previous reports in cats with confirmed FIP [14,17,19,24,25]. After treatment, one cat (JL) was identified to have hydrocephalus and syringohydromyelia, which has been associated with FIP [25,26]. It is unusual for cats under or following successful treatment to develop de novo neurological signs [16], so for our population, we hypothesize that the low dose of GS-441524 administered in combination with immunosuppressive retroviral coinfection was permissive to viral persistence in the nervous system. Alternative possibilities include unrelated neurologic disease, toxin exposure, and seizures unrelated to FIP. Three cats (18%) had ocular FIP, which is tentatively similar to the 5.5–13.1% previously described for cats with a confirmed diagnosis of effusive FIP [14,19,25].

Exploratory results analyzing disease characteristics, age, and chronology could suggest that there are differences among cats that are infected with retroviruses and those that are not, though the small sample size limits power and the ability to draw strong conclusions. The cats in this study were older upon suspected development of FIP than previously reported [14,17,19,24,25], likely because of our inclusion criteria of retroviral infection. Specifically, cats with FIV alone were significantly older upon diagnosis of FIP than cats with FeLV alone as previously reported [20]. Additionally, the interval between diagnosis of FIV and development of FIP was longer than that for FeLV when cats that were diagnosed with a retrovirus at the same time as FIP were excluded. A period of 35 months between diagnosis of FIV and development of FIP has been reported [27]. This is in contrast to a report of two historically FIV-positive cats who developed FIP within ten weeks of infection with FCoV [10]. The discrepancy may be due to all cats in the latter study being in the mid-to-late stages of FIV infection.

The potential difference in the age of development of FIP between FeLV and FIV populations could be related to the typical demographics affected: cats tend to be infected with FeLV at a younger age, often as kittens, whereas cats are usually infected with FIV as adults [28]. On the other hand, while strong conclusions about the biological behavior of these diseases are not possible to make given the limitations of our study, if the potential relationships shown here are representative, this may also suggest that there could be inherent differences between the two retroviral infections in immunosuppression or immune dysregulation and their effect on the development of FIP. Immunosuppression from FIV occurs late in life [6,10,29], and many cats with FIV experience a normal lifespan [9,30,31,32]. In contrast, FeLV is known to be a more pathogenic virus than FIV in domestic cats. Survival of cats with a high FeLV viral burden averages less than two years after diagnosis; however, cats with a low or regressive FeLV burden may live normal lifespans [8,33]. Quantitative PCR can assess the proviral load, which has been shown to be a reliable predictor of long-term survival of FeLV-infected cats [34]. As this diagnostic was not performed for any cat in this study, it remains unknown if the FeLV viral load influenced outcomes such as relapse and the development of neoplasia.

Conjectures about the interplay between immune function, retrovirus infection, and development of FIP can be further contemplated when observing the co-infected cats. In our report, the two FIV/FeLV co-infected cats were much older than cats that were reported to develop FIP with FeLV alone. Yet the time between diagnosis of a retrovirus and suspected development of FIP was much closer to the time for that of FeLV alone than FIV alone (1.5 and 2.7 months for co-infected cats compared to a median of 2.2 for FeLV-only and 34 for FIV-only). Consideration must be given to the fact that with natural retroviral infection, it is difficult to identify the chronicity of the infection as the FIV and FeLV infections in many of these cats were identified incidentally. One of the two FIV/FeLV co-infected cats (QIL) was diagnosed upon adoption. The cat’s age was estimated to be three years old at that time, so while this cat could have developed FIP within months of diagnosis of the retroviruses, the time of retroviral infection could still have been several years prior. On the other hand, the other cat (PIL) was diagnosed with FeLV after known exposure to an FeLV-positive cat and was diagnosed with FIP soon after. The preliminary data on age and disease characteristics discussed here may represent significant differences in presentation of FIP for each retrovirus; however, additional studies with a greater sample size are needed to draw meaningful conclusions. However, if this data is representative, it could be useful in the formation of differential diagnoses by veterinarians and monitoring of clinical signs throughout the life stages of retrovirus-positive cats by caretakers.

In this population, six cats underwent therapy for longer than 12 weeks. Dosages were increased for 11 cats throughout the entire treatment period, with greater magnitude in cats suspected to have neurologic and ocular FIP. Current protocols recommend a higher starting dose, especially in neurologic and ocular FIP cats, with dose escalation being associated with better outcomes and fewer relapses [22,23,35]. Thus, the high number of cats in this population that needed higher dosages, especially those diagnosed with neurologic and ocular FIP, is unsurprising. Data should be interpreted in light of the caretaker-reported, rather than objectively documented, dosages and durations, where caretakers are non-professionals reporting veterinary diagnostic information, and where, without availability of medical records, the data was not independently verified.

The overall prognosis for a response to therapy for presumptive FIP in this study was good, though overall survival was influenced by FeLV-associated disease. Four of seventeen cats in this study were deceased at the time of the survey, but most of the deaths were due to neoplasia and were therefore not suspected to be a direct result of FIP. Overall, most mortality was secondary to neoplasia. This is unsurprising as cats with FeLV and FIV are at a much greater risk of developing lymphoma or leukemia [6], and most FeLV-infected cats succumb to anemia or neoplasia [8].

Three of seventeen cats (18%) were thought to have relapsed but had survival of at least one year after the observation period. This is similar to other published relapse rates [12,14,15,17,36]; however, relapse rates herein relied on self-reported data that was not independently verified, so comparisons are tentative. In fact, two of the cats (JL, OL) did not have veterinary involvement throughout the course of treatment, but relapse was suspected in both due to the presence of clinical signs. All three of the cats that relapsed had FeLV. A recent study found that a greater proportion of cats with FeLV (11%) relapsed than cats without FeLV (6%), but this did not reach statistical significance [19]. This may be further evidence to support a different pathogenesis for FIP in cats with FeLV than FIV. In this population, beyond one-year survival, caution should be used when referencing overall survival data as exact dates of death were only available for a minority of cats.

This study has several limitations. These data were compiled from an owner survey rather than veterinary medical records, so objective data about the diagnosis and staging were not available. Six cats were considered “low confidence” based on a limited diagnostic work-up, and an additional respondent reported that a diagnosis was not made by a veterinarian. Inclusion of only effusive cases was intended to limit the possibility that the cats included in this study did not have FIP. Subsequent resolution of that effusion with antiviral therapy supported the presumptive diagnosis of FIP, though concurrent therapies could have led to resolution of other infections and inflammatory processes, limiting this interpretation and potentially influencing outcome statistics. A core limitation is that there was lack of information on the infection type of FeLV-positive cats (e.g., progressive, regressive, abortive, or focal), and the proviral load via quantitative PCR. This information would have allowed for better understanding of survival data for FeLV-infected cats [34]. Several sampling limitations exist that affect the representativeness of the population. Inclusion criteria and survey distribution methods limited the sample size. Accuracy is limited by recall bias; i.e., the accuracy of answers could be affected by prolonged amounts of time between the survey response and treatment or by limited understanding of the diagnostic and treatment process. For example, estimated dates sometimes relied on a generalized description given by respondents (e.g., “over a year”); however, a conservative approach was used in estimation so the data presented still has value as the overall survival may have been underestimated. Nevertheless, as a whole, this study may overestimate the effectiveness relative to a full clinical population. Additionally, the populations to which this survey was distributed and those who responded may not provide a representative sample of the circumstances of all FIP cats. For example, this convenience sample was developed from cat caretakers in social media groups dedicated to FIP treatment; it is possible that those with positive outcomes may be more likely to remain in the groups and submit the survey. Adjacently, several respondents’ cats were adopted while known to be infected with a retrovirus, and several cats were under the care of a shelter. The treatment decisions made by these demographics may not be representative of caretakers in general. These sampling limitations may affect the ability to accurately predict the treatment response and survival in a broader population. Finally, the initial survey was conducted in 2022–2023, prior to the availability of a validated source of GS-441524 and expert veterinary guidance in the USA. Thus, the outcomes presented here reflect an early-era, non-standardized treatment environment with variable product quality and dosing. Extrapolation of these results to current protocols should be made with caution.

Despite these limitations, this report suggests that treatment responses in some retrovirus-positive cats with presumptive FIP may be comparable to previously reported outcomes for FIP alone. This report may contribute to recent evidence that treatment of FIP in FeLV-positive cats can be highly successful both in the short term for most cats and for a subset of long-term survivors with FeLV [19] and suggests that this may also be possible for FIV-positive cats. This is particularly relevant given the new availability of compounded antiviral therapy for FIP in the United States. These findings suggest that retroviral coinfection alone should not automatically preclude consideration of antiviral treatment, although the prognosis may still be influenced by concurrent retrovirus-associated disease. This report also provides evidence that there may be differences in the presentation of FIP for each retrovirus, such as the age of development of FIP and the interval after retrovirus infection, which could be further investigated. Additional studies are needed in populations with a confirmed FIP diagnosis to determine ideal antiviral dosing regimens and durations of treatment for a rapid resolution of FIP and reduction in relapse in retrovirus-positive cats. In addition, the impact of factors such as the FeLV proviral load on the response to FIP therapy and long-term survival should be determined to provide caregivers with more complete prognostic information while FIP treatment is under consideration.

## Figures and Tables

**Table 1:** Clinical characteristics and chronology of feline infectious peritonitis (FIP), feline leukemia virus (FeLV), and feline immunodeficiency virus (FIV), as well as the method of calculation of survival dates (date type).

ID	FIV (I)	FeLV (L)	Effusion Location	Neurologic FIP	Ocular FIP	Age at FIP Diagnosis in Months	Months Between Diagnosis of FIV and FIP	Months Between Diagnosis of FeLV and FIP	Survival in Months	Date Type	Cause of Death
AI	+		A			154.7	40.1		9.3–28.2	L *	Unspecified
BI	+		T			9.2	5.6		13.0	L *	Unknown status
CI	+		A			7.5	0		42.6	L	Alive at time of follow-up
DI	+		A			267.3	27.9		40.6	L	Alive at time of follow-up
EI	+		A, P, T			165.7	115.5		7.7	S	Lymphoma, B-cell (jejunal mass)
FI	+		A	+ ^		65.8	45.4		44.4	L	Alive at time of follow-up
GL		+	A	+ ^		5.4		1.3	32.8	L	Alive at time of follow-up
HL		+	A			11.5		11.5	2.6	X	Acute lymphocytic leukemia
IL		+	T			38.7		2.2	40.0	L	Alive at time of follow-up
JL		+	A	+	+	8.0		3.1	28.2	X	Multifactorial, including chronic eosinophilic leukemia
KL		+	A			7.8		7.2	15.9	S	Unspecified
LL		+	A			4.5		4.0	45.3	L	Alive at time of follow-up
ML		+	T		+	43.7		0	52.8	L	Alive at time of follow-up
NL		+	A	+	+	8.2		0.8	28.0	L *	Unknown status
OL		+	A	+ ^		5.9		0	23.4	S	Lymphoma
PIL	+	+	A			120.2	10.6	1.5	24.3	S	Gastric neoplasia
QIL	+	+	A			39.2	2.7	2.7	44.2	S	Multifactorial, including lymphoma

A: abdominal effusion, FeLV: feline leukemia virus, FIP: feline infectious peritonitis, FIV: feline immunodeficiency virus, L: last known date alive, P: pericardial effusion, S: estimated date of death, T: thoracic effusion, X: exact date of death known; * Lost to follow-up; + Diagnosis present; ^ Developed neurologic signs after the start of GS-441524 therapy.

**Table 2:** The median and range of disease chronology for FeLV-only and FIV-only cats.

	FeLV	FIV
Age at diagnosis of retrovirus (months)	4.9 (0–43.7)	35.3 (3.6–239.4)
Age at diagnosis of FIP (months)	8.0 (4.5–43.7)	110.3 (7.5–267.3)
Months between retrovirus and FIP	2.2 (0–11.5)	34 (0–115.5)

**Table 3:** The most frequently reported clinical signs around the time of FIP diagnosis.

Clinical Feature	Number	Percentage
Hyporexia/anorexia	15	88%
Lethargy/listlessness	14	82%
Weight loss	10	59%
Distended abdomen	10	59%
Hiding/lack of socialization	7	41%
Difficulty breathing	7	41%
Difficulty walking/jumping	7	41%
Fever	7	41%
Pale gums	5	29%

**Table 4:** Diagnostics performed in each cat.

		AI	BI	CI	DI	EI	FI	GL	HL	IL	JL	KL	LL	ML	NL	OL	PIL	QIL
	CBC	●	●		●	●	●	●	●	●	●	●		●	●	●	●	●
	Chemistry	●	●	●	●	●	●	●	●		●			●	●	●		●
Imaging																		
	Abdominal Ultrasound	●		●		●	●	●	●	●	●		●					
	Radiographs		●							●	●			●			●	●
FIP Testing and Effusion Analysis																		
	Effusion Gross Examination	●	●	●	●	●	●		●	●	●	●	●	●	●	●	●	●
	Effusion Complete Fluid Analysis			●			●			●	●			●				
	Effusion Cytology			●		●			●		●			●				
	Effusion Rivalta	●												●			●	
	FCoV RT-PCR														●			●
	FIP mRNA PCR					●												
	FIP ELISA 7b Protein			●					●									
	FIP Virus RealPCR			●					●									
	FCoV Antibody Titer													●			●	
	FCoV IFA Antibody								●									
FIV Testing																		
	POC Antibody		●	●	●	●	●										●	●
	PCR	●																
	Western Blot Antibody					●												
FeLV Testing																		
	POC p27 Antigen							●	●	●	●	●	●	●	●	●	●	●
	PCR							●	●		●			●	●		●	●
	IFA p27 Antigen										●	●	●		●		●	

●: diagnostic was performed; CBC: complete blood count; ELISA: enzyme-linked immunosorbent assay; IFA: immunofluorescence assay; POC: point of care; PCR: polymerase chain reaction.

**Table 5:** Treatment details for cats that relapsed, including the length of treatment and time until a subsequent relapse.

ID	Initial Therapy Length in Weeks	Weeks to First Relapse	Additional Therapy Length	Weeks to Second Relapse	Additional Therapy Length	Total Weeks to Completion	Start Dosage	End Dosage
JL	12	<1	24	NA	NA	36	4 mg/kg/d SC	18 mg/kg/d PO
ML	12	1	5	1	7	26	8 mg/kg/d SC	25 mg/kg/d PO
OL	12	4	6	NA	NA	22	8 mg/kg/d SC	10 mg/kg/d SC

PO: per os; SC: subcutaneous; NA: not applicable.

**Table 6:** The starting and ending dosage for all cats with data available (*n* = 14), and for neurologic (starting and all time) or ocular FIP.

Dosage (mg/kg/d)		All	Neurologic at Diagnosis	All Neurologic	Ocular FIP
Start (Injectable)	N	14	2	5	3
	Average	6.8	8.0	7.6	8.0
	Range	1.8–12.0	4.0–12.0	4.0–12.0	4.0–12.0
End (Injectable)	N	10	1	4	1
	Average	7.1	12.0	8.9	25.0
	Range	2.5–12.0	-	3.4–12.0	-
End (Oral)	N	4	1	1	2
	Average	15.3	18.0	18.0	15.0
	Range	8.0–25.0	-	-	12.0–18.0

N: number of cats.

## Data Availability

The data presented in this study are available on request from the corresponding author to protect respondent privacy.

## Supplementary Materials

The following supporting information can be downloaded at: https://www.mdpi.com/article/10.3390/pathogens15030337/s1, File S1: FIP Qualtrics Survey Questions.

## Abbreviations

The following abbreviations are used in this manuscript:

FCoV	Feline Coronavirus
FeLV	Feline Leukemia Virus
FIP	Feline Infectious Peritonitis
FIPV	Feline Infectious Peritonitis Virus
FIV	Feline Immunodeficiency Virus
