# Supplementary material for: Clinical Features and Outcomes of Treatment for Effusive Feline Infectious Peritonitis with GS-441524 in Seventeen Retrovirus-Positive Cats
Source: Pathogens. 2026 Mar 21;15(3):337. doi: 10.3390/pathogens15030337 (PMC13029278; doi:10.3390/pathogens15030337)
Supplement: Supplementary file 1 [file pathogens-15-00337-s001.zip › FIP Qualtrics Survey Questions pg.pdf]

Informed Consent, Patient Signalment

The Ohio State University Consent to Participate in Research

Study Title: FIP Treatment Survey for Cats with FeLV/FIV

Protocol Number: 2021E0162

Researcher: Samantha J.M. Evans, DVM, PhD, DACVP

Sponsor: EveryCat Foundation

This is a consent form for research participation. It contains important information about this study and what to expect if you decide to participate. It contains important information about this study and what to expect if you decide to participate.

Your participation is voluntary.

Please consider the information carefully. Feel free to ask questions before making your decision whether or not to participate.

Purpose: We are researching the use of a unlicensed antiviral therapeutic compound known as GS-441524 for treatment of feline infectious peritonitis (FIP).

Procedures/Tasks: Cat owners who have used this unlicensed drug to treat their cats will take a single online survey.

Duration: The surveys will take approximately 20-25 minutes to complete.

You may leave the study at any time. If you decide to stop participating in the study, there will be no penalty to you, and you will not lose any benefits to which you are otherwise entitled. Your decision will not affect your future relationship with The Ohio State University.

Risks and Benefits: There are no direct benefits to the participants, other than helping us to learn more about what factors determine success for future cats. There are no risks to participants.

Confidentiality:

We will work to make sure that no one sees your online responses without approval. But, because we are using the Internet, there is a chance that someone could access your online responses without permission. In some cases, this information could be used to identify you.

Also, there may be circumstances where this information must be released. For example, personal information regarding your participation in this study may be disclosed if required by state law. Also, your records may be reviewed by the following groups (as applicable to the research):

- Office for Human Research Protections or other federal, state, or international regulatory agencies
- The Ohio State University Institutional Review Board or Office of Responsible Research Practices

No such breach of confidentiality is expected.

Future Research: Your de-identified information may be used or shared with other researchers without your additional informed consent.

Incentives: There are no financial incentives for participating in this research.

Participant Rights:

You may refuse to participate in this study without penalty or loss of benefits to which you are otherwise entitled. If you are a student or employee at Ohio State, your decision will not affect your grades or employment status.

If you choose to participate in the study, you may discontinue participation at any time without penalty or loss of benefits. By agreeing to participate, you do not give up any personal legal rights you may have as a participant in this study. This study has been determined Exempt from IRB review.

Contacts and Questions:

For questions, concerns, or complaints about the study you may contact Dr. Samantha Evans at evans.2608@osu.edu .

For questions about your rights as a participant in this study or to discuss other study-related concerns or complaints with someone who is not part of the research team, you may contact the Office of Responsible Research Practices at 1-800-678-6251 or hsconcerns@osu.edu.

Providing consent

I have read (or someone has read to me) this page and I am aware that I am being asked to participate in a research study. I have had the opportunity to ask questions and have had them answered to my satisfaction. I voluntarily agree to participate in this study. I am not giving up any legal rights by agreeing to participate.

To print or save a copy of this page, select the print button on your web browser.

Please select "I agree" below to proceed and participate in this study. If you do not wish to participate, please select "I disagree" or close out your browser window. "

- ○ I agree
- ○ I disagree

What is your email address?

____

In which country do you currently reside?

[▾]

What is your cat's name?

(Please enter survey information for only one cat at a time. A separate survey must be completed for each cat within the household that is enrolled in this study.)

What is your cat's breed? (Please check all that apply)

- [ ] Domestic Mixed-Breed/"Moggie"
- [ ] Abyssinian
- [ ] American Bobtail
- [ ] American Curl
- [ ] American Shorthair
- [ ] American Wirehair
- [ ] Balinese
- [ ] Bengal
- [ ] Birman
- [ ] Bombay
- [ ] British Shorthair
- [ ] Burmese
- [ ] Burmilla
- [ ] Chartreux
- [ ] Chausie
- [ ] Colorpoint Shorthair
- [ ] Cornish Rex
- [ ] Devon Rex
- [ ] Egyptian Mau
- [ ] European Burmese
- [ ] Exotic
- [ ] Havana Brown
- [ ] Himalayan
- [ ] Japanses Bobtail
- [ ] Khao Manee
- [ ] Korat
- [ ] Lykoi
- [ ] Maine Coon
- [ ] Manx
- [ ] Norwegian Forest Cat
- [ ] Ocicat
- [ ] Oriental
- [ ] Persian (including Himalayan)
- [ ] RagaMuffin
- [ ] Ragdoll
- [ ] Russian Blue
- [ ] Savannah
- [ ] Sottish Fold
- [ ] Selkirk Rex
- [ ] Serengeti
- [ ] Siamese
- [ ] Siberian
- [ ] Singapura
- [ ] Somali
- [ ] Sphynx
- [ ] Tonkinese
- [ ] Toybob
- [ ] Toyger
- [ ] Turkish Angora
- [ ] Turkish Van
- [ ] Other: (please specify)
- [ ] Unknown/not sure

☐ LaPerm

What is your cat's date of birth (or your best estimate)?

	Month	Day	Year
Please Select:			

What was your cat's sex and reproductive status at the time of their FIP diagnosis?

- ○ Male, Intact
- ○ Male, Neutered
- ○ Female, Intact
- ○ Female, Spayed

At time of diagnosis and during treatment, who had ownership of the cat?

- ○ Private ownership - personal pet
- ○ Rescue group/shelter
- ○ ________ Other (please specify)

Did a veterinarian diagnose this cat with FIP?

- ○ Yes, my own veterinarian
- ○ Yes, the veterinarian from the shelter/rescue
- ○ No, a vet ran tests on the cat, but the cat was diagnosed online using the diagnostic results from the vet
- ○ No, a non-veterinarian from the shelter/rescue made the diagnosis
- ○ No, the cat was diagnosed by a non-veterinarian through the shelter/rescue group based on clinical signs and lab tests

- ○ [ ] Other (please specify)

At the time of FIP diagnosis, how long had you had your cat for? (Adopted or fostered).

- ○ Less than 1 month
- ○ Between 1 month and 6 months
- ○ Between 6 months and 1 year
- ○ Between 1 year and 2 years
- ○ Between 2 years and 5 years
- ○ 5 years or more

Where/what situation did you adopt your cat from?

- ○ Shelter
- ○ Rescue group
- ○ Breeder
- ○ Pet Store
- ○ Found as a stray
- ○ Gift from a friend or family member
- ○ A kitten from my own cat's litter
- ○ Re-homing from a stranger
- ○ [ ] Other: (please specify)

What was your cat's weight at the beginning of treatment?

Please specify weight in pounds ONLY. If you do not know your cat's weight, please enter "0".

Please provide weight in **decimals only**, NOT ounces (eg. 8.5 pounds instead of 8 pounds 8 ounces). **Only numeric responses with up to two decimals will be accepted.**

To convert weight from kilograms to pounds, multiply weight in kilogram by 2.2 (pounds (lbs.) = kilograms (kg.) x 2.2).

What was your cat's weight at the end of treatment?

Please specify weight in pounds ONLY. If you do not know your cat's weight, please enter "0".

Please provide weight in **decimals only**, NOT ounces (eg. 8.5 pounds instead of 8 pounds 8 ounces). **Only numeric responses with up to two decimals will be accepted.**

To convert weight from kilograms to pounds, multiply weight in kilogram by 2.2 (pounds (lbs.) = kilograms (kg.) x 2.2).

FIP Dx Date

What was the date of your cat's FIP diagnosis?

	Month	Day	Year
Please Select:			

FIP Specific/Medical and Txs

What type(s) of FIP was your cat diagnosed with? (Please check all that apply)

- ☐ Effusive (wet)
- ☐ Non-effusive (dry)
- ☐ Neurological
- ☐ Ocular
- ☐ Unknown/Not Sure

Was there effusion/fluid present when the cat was diagnosed with FIP? The fluid could have been in the abdomen, in the chest, or around the heart.

- ○ Yes
- ○ No

Was the fluid collected for testing or to improve the cats comfort?

- ○ Yes
- ○ No

Please check all descriptions that apply to the fluid that was collected. There may be more than one answer.

- ☐ Yellow tinged fluid
- ☐ Green tinged fluid
- ☐ Clear fluid
- ☐ Cloudy fluid
- ☐ Red/pink tinged fluid
- ☐ Viscous, sticky or thick fluid
- ☐ High protein fluid
- ☐ Purulent

- [ ] Transudate
- [ ] Modified transudate
- [ ] Inflammatory exudates
- [ ] Bacterial peritonitis or fluid consistent with sepsis
- [ ] I do not know

Did YOU observe your cat experiencing any of the following symptoms around the time of diagnosis? (Please check all that apply)

- [ ] Anisocoria (different sized-pupils)
- [ ] Blindness
- [ ] Bloody stool
- [ ] Color changes or spots in the eye
- [ ] Constipation/obstipation (difficulty defacating)
- [ ] Cough
- [ ] Decreased appetite or refusal to eat
- [ ] Diarrhea
- [ ] Difficulty breathing
- [ ] Difficulty walking or jumping
- [ ] Distended abdomen (swollen belly due to fluid build-up)
- [ ] Hiding/lack of socialization
- [ ] Incontinence: fecal (loss of ability to control bowel movements)
- [ ] Incontinence: urinary (loss of bladder control)
- [ ] Increased water consumption
- [ ] Increase in urinations (volume and/or frequency)
- [ ] Jaundice (yellowed skin or eyes)
- [ ] Lethargy/listlessness
- [ ] Pale gums
- [ ] Paralysis (partial or total)
- [ ] Pica (eating/licking of inappropriate objects, eg. platic bags, paper, kitty litter)
- [ ] Seizures (either focal or generalized)

- [ ] Tremors or shaking
- [ ] Upper respiratory issues (ie. sneezing, congestion, nasal discharge)
- [ ] Vocalization
- [ ] Vomiting
- [ ] Weight loss
- [ ] ______ Other: (please specify)
- [ ] No symptoms/issues were observed

Around the time of your cat's FIP diagnosis, was your cat diagnosed as having any of the following conditions by a veterinarian? (Please check and answer all that apply)

- [ ] ______ Ocular (eye) involvement (please describe)
- [ ] ______ Oral (mouth) involvement (please describe)
- [ ] Cardiac (heart) involvement/disease (please describe) ______
- [ ] ______ Lower (lung) respiratory disease (please describe)
- [ ] ______ Upper respiratory disease (please describe)
- [ ] Upper urinary tract (kidney) involvement (please describe) ______
- [ ] Lower urinary tract (bladder) involvement (please describe) ______
- [ ] ______ Hepatic (liver) involvement (please describe)
- [ ] Neurological involvement/disease (please describe) ______
- [ ] Gastrointestinal tract involvement (please describe) ______
- [ ] ______ Bone marrow involvement (please describe)
- [ ] Granulomas: intestinal (inflammatory nodules in the intestines)
- [ ] Granulomas: lung (inflammatory nodules in the lungs)
- [ ] Lymphoma

- [ ] Enlarged lymph nodes
- [ ] Fluid in the abdomen (in the belly)
- [ ] Fluid in the chest (around the lungs)
- [ ] Fluid in the pericardium (around the heart)
- [ ] Fever
- [ ] Mediastinal mass
- [ ] Stomatitis (inflamed gums)
- [ ] Weight loss
- [ ] ____________ Other: (please specify)
- [ ] No other medical conditions were diagnosed by my veterinarian

Which of the following diagnostics were performed to help diagnose FIP? (Please check all that apply)

- [ ] Abdominal ultrasound
- [ ] Biopsy with IHC (immunohistochemical staining)
- [ ] Biopsy without IHC (immunohistochemical staining) - histopathology only
- [ ] CBC (complete blood count; a common type of blood test)
- [ ] Chemistry panel (AKA biochemical profile; a common type of blood test)
- [ ] CT (AKA "cat scan")
- [ ] Cytology (via fine needle aspirate)
- [ ] ECG/EKG (electrocardiogram)
- [ ] Feline coronavirus (FCoV) antibody titer
- [ ] Feline coronavirus (FCoV) RT-PCR
- [ ] FIP ELISA 7b protein test (by Antech Laboratories)
- [ ] FIP IFA (immunofluorescence assay)
- [ ] FIP mRNA PCR
- [ ] FIP Virus RealPCR test (by IDEXX Laboratories)
- [ ] Fluid analysis: cerebrospinal fluid (CSF) from a spinal tap
- [ ] Fluid analysis: complete fluid analysis of effusion fluid
- [ ] Fundic exam (a specific type of eye exam)

- [ ] Neruological exam
- [ ] MRI
- [ ] Radiographs (x-rays)
- [ ] Rivalta test (test conducted on effusion fluid)
- [ ] ____________ Other: (please specify)
- [ ] None - no specific diagnostic tests were performed by my veterinarian

Other than FIP and FeLV and/or FIV, does your cat have any other concurrent illnesses?
If yes, please write in the box below and specify if it was diagnosed before or after their FIP diagnosis.

____

During the course of GS-441524 therapy, did your cat receive any of the following medications or treatments other than GS-441524, including those administered directly by your veterinarian (eg. an antibiotic injection)? (Please check all that apply)

- [ ] ____________ Antibiotics (please specify type)
- [ ] Blood transfusion
- [ ] Anti-nausea medications (eg. cerenia, maropitant, ondansetron)
- [ ] Injectable steroids (eg. depomedrol, dexamethasone)
- [ ] Gabapentin/neurontin
- [ ] Fluid administration: subcutaneous (SC/SQ)
- [ ] Fluid administration: intravenous (IV)
- [ ] NSAID (eg. meloxicam, metacam, onsior)
- [ ] Oxygen therapy
- [ ] Polyprenyl Immunostiumlant (PI) or VetImmune
- [ ] Oral steroids (eg. prednisolone): (please specify dose and frequency) ____________

- [ ] T-cyte/Proboost/Thymic Protein A supplementation
- [ ] Vitamin B12 injections
- [ ] ______ Other: (please specify)
- [ ] No medications were received/administered

During the course of GS-441524 therapy, did your cat receive any of the following supplemental antiviral therapies? (Please check all that apply)

- [ ] Chloroquine
- [ ] Cyclosporine A
- [ ] GC376
- [ ] Itraconazole
- [ ] Mefloquine
- [ ] Molnupiravir (EIDD-2801)
- [ ] Remdesivir
- [ ] ______ Other (please describe)
- [ ] No, my cat did not receive any of these therapies

GS Chronology and Dosing, Relapse

What was the date when you **began** treatment with GS-441524 for you cat?

	Month	Day	Year
Please Select:			

What was the date when you **finished** treatment with GS-441524 for your cat? If your cat is still receiving treatment, please input today's date instead.

	Month	Day	Year
Please Select:			

What form of GS-441524 therapy did you use?

- ◯ Injectable only
- ◯ Oral/pills only
- ◯ Combination of both injections and oral/pills

What was your **starting** dosage of GS-441524?

This would have been how many mg (milligrams) per kg (kilograms) of body weight your cat was given at the beginning of treatment (ex: 4 mg/kg).

How frequently was GS-441524 given when you started treatment?

- ◯ Once a day/every 24 hours
- ◯ Twice a day/every 12 hours
- ◯ Three times a day/every 8 hours
- ◯ Other

What form of GS-441524 was given when your cat first started treatment?

- ◯ Injectable
- ◯ Oral
- ◯ Both injectable and oral

What was your **ending** dosage of GS-441524?

This would have been how many mg (milligrams) per kg (kilograms) of body weight your cat was given at the end of treatment (ex: 6 mg/kg).

How frequently was GS-441524 being given at the end of treatment?

- Once a day/every 24 hours
- Twice a day/every 12 hours
- Three times a day/every 8 hours
- Other

What form of GS-441524 was being given to the cat at the end of treatment?

- Injectable
- Oral
- Both injectable and oral

(Optional) Please describe any dosing regimen changes you remember implementing and why you decided to make those changes (ex. going from once daily to twice daily, changing formulation from liquid to pills, changing brands, temporary dosing increases/decreases, etc).

What phase of FIP treatment is your cat currently in?

- ○ Unfortunately, my cat passed away during GS-441524 treatment
- ○ Unfortunately, my cat passed away after finishing therapy with GS-441524
- ○ My cat is currently in the first 12 weeks of therapy with GS-441524
- ○ My cat is currently in an extended period of treatment following the initial 12 weeks of therapy with GS-441524
- ○ My cat is currently in the 12 week observation period
- ○ My cat has finished the observation period (following GS therapy and 12 weeks of observation)
- ○ My cat has finished the observation period (following GS therapy and 12 weeks of observation) over 1 year ago
- ○ ________ Other

For how many **weeks** was your cat treated with GS-441524 therapy? (Ex: 12 weeks)

If your cat relapsed and underwent a second round of treatment, please include the number of weeks your cat was treated for during the first round of treatment only.

Please provide your answer rounded to the nearest number of weeks. This question accepts numeric answers only.

____

Did you extend the treatment period for GS-441524 therapy past the 12 weeks period? (Please check all that apply)

- ☐ No, my cat was treated for 12 weeks or less
- ☐ Yes, because my cat's blood work indicated that he/she should be treated longer
- ☐ Yes, because my cat was still showing clinical signs of FIP

- ☐ Yes, to be extra cautious for general reasons
- ☐ Yes, to be cautious because my cat also has FeLV
- ☐ Yes, to be cautious because my cat also has FIV
- ☐ ________ Other: (please specify)

Did your cat suffer a relapse of clinical signs of FIP?

- ○ No
- ○ Yes

How long after the initial treatment ended did the relapse occur?

Please answer in numbers of weeks (ie. enter "2" for 2 weeks). If the relapse occurred less than one week after the end of treatment, please enter "0". Only numeric answers will be accepted.

____

How long did you administer relapse treatment for?

Please answer in numbers of weeks (ie. enter "2" for 2 weeks). Only numeric answers will be accepted.

____

Did you or your admin make any changes in dosing or frequency given during the relapse treatment period? If yes, please explain.

- ○ No
- ○ Yes (please describe dose change and the reason for dose change): ________

Were there any additional relapses after the previously mentioned relapse? If yes, please explain how long after treatment was ended and how long you continued the third treatment for (in weeks).

- ○ No
- ○ Yes

Please explain the timeline of the additional relapse(s), including when the additional relapse(s) occurred and the duration of the additional treatment(s).

How soon after beginning treatment with GS-441524 did you **notice improvements in your cats clinical signs** associated with FIP? (Choose the closest answer, even if it is not exact.)

- ○ Within the first 24 hours
- ○ Within the first 48 hours
- ○ Within the first 72 hours
- ○ About 1 week
- ○ About 2 weeks
- ○ About 4 weeks/1 month
- ○ About 6 weeks
- ○ About 8 weeks/2 months
- ○ About 12 weeks
- ○ Longer than 12 weeks

- My cats clinical signs never improved

How soon after beginning treatment with GS-441524 did your cat **return to fully normal behavior?** (Choose the closest answer, even if it is not exact.)

- Within the first 24 hours
- Within the first 48 hours
- Within the first 72 hours
- About 1 week
- About 2 weeks
- About 4 weeks/1 month
- About 6 weeks
- About 8 weeks/2 months
- About 12 weeks
- Longer than 12 weeks
- My cat never returned to fully normal behavior : (please describe)

FIV, FeLV, or Both

Has your cat tested positive for FeLV (feline leukemia virus), FIV (feline immunodeficiency virus), or both?

- FeLV only
- FIV only
- FeLV and FIV both
- Neither FeLV nor FIV

FelV Questions

Between FeLV and FIP, what was your cat diagnosed with first?

- ◯ FIP
- ◯ FeLV
- ◯ Diagnosed with both diseases at the same time

What was the approximate month and year of your cat's FeLV diagnosis?

	Month	Year
Please Select:		

How did you find out this cat had FeLV?

- ◯ We tested the cat at our shelter/rescue
- ◯ The shelter/rescue told me this cat was positive when they were adopted
- ◯ My personal vet tested as part of routine wellness care
- ◯ My personal vet tested when my cat was sick or injured
- ◯ Diagnosed when he/she went in to be neutered/spayed
- ◯ Diagnosed when the cat began to have clinical signs of FIP
- ◯ ______ Other: (please specify)

What test(s) were used to diagnose your cat with FeLV? (Please check all that apply)

- ☐ Rapid in-house test at my veterinarian; or at the shelter/rescue
- ☐ Immunofluorescent assay (IFA) (sent out to a laboratory)
- ☐ FeLV PCR Test (sent out to a laboratory)
- ☐ ______ Other: (please specify)
- ☐ Unsure

Was your cat ever vaccinated for FeLV?

- ○ Yes
- ○ No
- ○ Unsure

What clinical signs, or secondary disorders did your cat have that were attributed to having FeLV (and not FIP)? (Please check all that apply)

- ☐ Abortion (loss of pregnancy)
- ☐ Acute lymphoid leukemia (ALL) (extremely high lymphocyte count)
- ☐ Anisocoria (pupils are different sizes - one small and one large)
- ☐ Anemia (low red blood cells)
- ☐ Anorexia (not wanting to eat)
- ☐ Bladder infection
- ☐ Blindness
- ☐ Bloody stool
- ☐ ________ Cancer - lymphoma: (please specify form/type)
- ☐ ________ Cancer - other than lymphoma: (please specify)
- ☐ Chronic lymphoid leukemia (CLL) (extremely high lymphocyte count)
- ☐ Conjunctivitis (redness and inflammation of the lining around the eyes)
- ☐ Diarrhea
- ☐ Enlarged lymph nodes
- ☐ Fever
- ☐ Gingivitis/stomatitis (red and inflamed gums)
- ☐ ________ Immune mediated disease: (please specify)
- ☐ Incontinence: fecal (loss of ability to control bowel movements)
- ☐ Incontinence: urinary (loss of bladder control)
- ☐ Lethargy/listlessness
- ☐ Neutropenia (low white blood cells)
- ☐ Ocular (eye) infection

- [ ] Oral ulcers
- [ ] Pale gums
- [ ] Seizures
- [ ] Skin infection
- [ ] Upper respiratory issues (ie. sneezing, congestion, nasal discharge)
- [ ] Vomiting
- [ ] Weight loss
- [ ] NONE of these clinical signs or conditions - only indication of FeLV is the positive test result
- [ ] ________ Other (please specify):

What treatments were utilized to treat FeLV or FeLV associated signs? (Please check all that apply)

- [ ] None
- [ ] Alpha-FeLV treatment
- [ ] Antibiotics
- [ ] Chemotherapy - various kinds to treat lymphoma
- [ ] Zidovudine (azidothymidine, AZT "Retrovir")
- [ ] Decitabine
- [ ] Feline Interferon Omega
- [ ] Gemcitabine
- [ ] Human Interferon Alpha
- [ ] LTCI by TCyte
- [ ] Nutracuticals/Supplements
- [ ] Raltegravir
- [ ] Steroids (such as prednisone)
- [ ] Suramin
- [ ] Tenofovir
- [ ] ________ Other: (please specify)

File Uploads

Please upload any veterinary records, test results, or other diagnostic data that were used as part of the diagnosis for your cat.

Only one file can be uploaded per slot. 8 upload slots are provided. If you have more than 8 files to share, please compress them into a zip file or email them to fipwarriorsstudy@gmail.com. Files must be less than 100 Mb in size. You do not need to utilize all 8 file upload slots.

File upload slot #1:

File upload slot #2:

File upload slot #3:

File upload slot #4:

File upload slot #5:

File upload slot #6:

File upload slot #7:

File upload slot #8:

Satisfaction

How satisfied are you with your experience of undergoing GS therapy for your cat?

- Very satisfied
- Satisfied
- Neutral
- Dissatisfied
- Very dissatisfied

In the future, if you have another cat diagnosed with FIP, how likely would you be to undertake GS therapy for them?

- Very likely
- Likely
- Neutral
- Unlikely

○ Very unlikely

Please elaborate on your answers to the previous two questions.

[ ]

What did your veterinarian tell you about treatment when your cat was diagnosed with FIP?

○ There was not curative treatment

○ There was a treatment, but it was not avaiable

○ There was a treatment and referred me to another organization (such as the Warriors) for more information

○ There was a treatment and my vet explained it in detail

○ ______ Other: (please specify)

How did you hear about GS-441524 therapy? (Please check all that apply)

☐ A friend or family member told me about it

☐ I found out about it while doing my own research: (please specify specific ______ source(s))

☐ I was already a member of the FIP Warriors Facebook group(s): (please ______ explain)

☐ I knew about it previously but not through the FIP Warriors Facebook group(s): ______ (please explain)

☐ My general practice veterinarian told me about it

☐ I was referred to a veterinary specialist who then told me about it: (please ______ specify)

- [ ] My shelter/rescue group knew about it

Did you have help from a veterinary professional during treatment with GS-441524?

- ○ No, I ordered the medication and treated my cat myself without the help of a veterinarian after the initial diagnosis
- ○ Yes, my vet was aware that I was providing GS-441524 therapy to my cat at home and monitored my cat throughout treatment and observation
- ○ My vet did recheck exams and blood work on my cat, but was not aware that I was giving my cat GS-441524 therapy at home
- ○ My veterinarian was extremely helpful and either my vet or their support staff administered GS-441524 therapy to my cat
- ○ My veterinarian supplied me with GS-441524 and played an active role in supporting my administration of GS-441524 therapy to my cat

Final Questions/comments

(Optional) If you would like to provide any questions, comments, or additional details about your cat that you think might be important to provide at this time, please include them here:

FIV Questions

Between FIV and FIP, what was your cat diagnosed with first?

- FIP
- FIV
- Diagnosed with both diseases at the same time

What was the approximate month and year of your cat's FIV diagnosis?

	Month	Year
Please Select:		

How did you find out your cat had FIV?

- Was told when the cat was adopted that he/she was FIV positive
- Diagnosed at a healthy/wellness visit
- Diagnosed following an illness or injury
- Diagnosed when he/she went in to be neutered/spayed
- Diagnosed when the cat began to have clinical signs of FIP
- Other: (please specify)

What test(s) were used to diagnose your cat with FIV? (Please check all that apply)

- POC (point of care) test: in-house rapid test
- PCR test - other (sent out to a laboratory)
- Western blot (sent out to a laboratory)
- Other: (please specify)
- Unsure

Was your cat ever vaccinated for FIV? (Note: this is NOT a core vaccine or a vaccine routinely given).

- ○ Yes
- ○ No
- ○ Unsure

What clinical signs, or secondary disorders did your cat have that were attributed to having FIV (and not FIP)? (Please check all that apply)

- ☐ Abortion (loss of pregnancy)
- ☐ Acute lymphoid leukemia (ALL) (extremely high lymphocyte count)
- ☐ Anisocoria (pupils are different sizes - one small and one large)
- ☐ Anemia (low red blood cells)
- ☐ Anorexia (not wanting to eat)
- ☐ Bladder infection
- ☐ Blindness
- ☐ Bloody stool
- ☐ ________ Cancer - lymphoma: (please specify form/type)
- ☐ ________ Cancer - other than lymphoma: (please specify)
- ☐ Chronic lymphoid leukemia (CLL) (extremely high lymphocyte count)
- ☐ Conjunctivitis (redness and inflammation of the lining around the eyes)
- ☐ Diarrhea
- ☐ Enlarged lymph nodes
- ☐ Fever
- ☐ Gingivitis/stomatitis (red and inflamed gums)
- ☐ ________ Immune mediated disease: (please specify)
- ☐ Incontinence: fecal (loss of ability to control bowel movements)
- ☐ Incontinence: urinary (loss of bladder control)
- ☐ Lethargy/listlessness
- ☐ Neutropenia (low white blood cells)
- ☐ Ocular (eye) infection
- ☐ Oral ulcers
- ☐ Pale gums

- [ ] Seizures
- [ ] Skin infection
- [ ] Upper respiratory issues (ie. sneezing, congestion, nasal discharge)
- [ ] Vomiting
- [ ] Weight loss
- [ ] NONE of these clinical signs or conditions - only indication of FIV is the positive test result
- [ ] ____________ Other (please specify):

What treatments were utilized to treat FIV? (Please check all that apply)

- [ ] Adefovir
- [ ] Antibiotics
- [ ] Chemotherapy - various kinds to treat lymphoma
- [ ] Feline Interferon Omega
- [ ] Human Interferon Alpha
- [ ] Lamivudine
- [ ] LTCI by TCyte
- [ ] Nutracuticals/Supplements
- [ ] Plerixafor
- [ ] Steroids (such as prednisone)
- [ ] Tenofovir
- [ ] Zidovudine (azidothymidine, AZT "Retrovir")
- [ ] ____________ Other: (please specify)
- [ ] No treatments were used to treat FIV
